# The Prevalence of Myopia Among Primary School Male Students in Bisha, Saudi Arabia

**DOI:** 10.7759/cureus.36792

**Published:** 2023-03-28

**Authors:** Ibrahim Eljack, Yazeed Alshahrani

**Affiliations:** 1 Community Medicine, College of Medicine, Bisha University, Bisha, SAU

**Keywords:** student, spherical equivalent, risk factors, prevalence, myopia

## Abstract

Background

Myopia (shortsightedness) is considered a major health problem globally which has increased in the last few decades. This study aims to determine the prevalence of myopia and the associated risk factors among primary school students in Bisha, Saudi Arabia.

Methods

This descriptive cross-sectional study included 330 male students from five boys’ schools in Bisha city. Students underwent an interview questionnaire that composed of (sociodemographic data, risk factors, and ocular history). Students’ vision was assessed by an optometrist through the use of a Snellen chart and the result was converted to a Diopter unit (D). Myopia was defined as the spherical equivalent (SE) of ≤ - 0.5 D. Binomial statistical test was used to get a prevalence of myopia with a confidence interval (CI) of 95%. Chi-square analytical test was used to compare myopic and non-myopic groups on multiple variables. Results were considered statistically significant at p-value ≤ 0.05.

Results

The mean age of 330 male students was 11.29 ± 0.97. The prevalence of myopia was (32.7%, 95% CI: 27.7-38.1%), and the mean of the SE of participants was - 0.25 ± 0.60 D. Myopia prevalence was increased with age and school grade of participants (p ≤ 0.05). Students who spent a long time (more than three hours) on near activity are at risk of developing myopia (p ≤ 0.001). having one or both parents affected by myopia was not statistically significant with the prevalence of myopia (p = 0.175). Children who spent a long time outdoors tend to have a lower risk of myopia (p ≤ 0.001).

Conclusion

The study showed a high prevalence of myopia among schoolchildren in Bisha city. Therefore, it is recommended to plan for future screening programs for myopia.

## Introduction

Myopia (shortsightedness) is an eye disorder characterized by the inability of the person to see further things clearly [1]. It is considered a benign disorder that can be corrected by either glasses, contact lenses, or refractive surgery [1]. However, myopia is one most common health issues around the world and its prevalence is increasing with each decade [2]. Although, it is a benign condition high grades of myopia increase the possibility of developing ocular pathological diseases such as cataracts, glaucoma, retinal detachment, and myopic macular degeneration which may result eventually in irreversible vision loss. In addition, it is considered to be the most frequent cause of irreversible blindness in some areas of the world [3].

Myopia is caused by the contribution of genetic factors and environmental factors. Concerning genetic factors, some studies showed that myopic parents develop the risk of having myopic children. On the other hand, environmental factors could be due to: (a) socioeconomic factors: some studies in china and japan showed that families with high income have a risk of getting myopia; (b) near work factors: it is mainly the excessive use of the eyes while studying, reading, watching television; (C) less outdoor activities [4,5].

Myopia is a prevalent health problem accounting for 22.9% or 1406 million people with myopia in the whole world [3]. A study has been done on schoolchildren in rural India reported that Myopia's prevalence is 39.6% [6]. Another study in Taif, Saudi Arabia reported a prevalence of myopia among schoolchildren to be 33.28% [7]. Myopia can make vision blurry, which can impair daily living activities if not detected and corrected early, which might also inversely affect students' educational performance. Regular screening sessions among students of schools and colleges can lead to minimizing the morbidity of myopia and enhance the awareness of the community towards periodic vision examinations and their importance.

## Materials and methods

A descriptive cross-sectional study was done on male students aged 10 to 13 years old in Bisha city located in the southern region of Saudi Arabia. The data was collected over a duration of one month in 2021 as part of a health campaign provided by Bisha college of medicine to screen primary schoolchildren for refractive errors in five different boys’ schools in Bisha. The sampling technique was a simple random sampling of students. Students with eye anomalies and systemic diseases that may affect vision status were excluded. The data collection process was through an interview questionnaire by trained medical students. The questionnaire was divided into four main domains sociodemographic data (age, school year, nationality, place of residence), risk factors for developing myopia (family history, duration of outdoor activities; time spent on reading and writing activities, and use of electronic devices), ocular history section (history of eye disease, eye symptoms, wearing spectacles) and last section for vision status (myopia or normal vision). The assessment of the eye for refractive errors was provided by optometrists by using the Snellen chart as a screening tool for myopia and then referred those with abnormal assessment results for further assessment by an autorefractor and to refer them to an optometrist or ophthalmologist when needed.

Data were analyzed using Statistical Package for the Social Sciences (IBM Corp. Released 2015. IBM SPSS Statistics for Windows, Version 23.0. Armonk, NY: IBM Corp). Descriptive data were expressed using frequencies, charts, and continuous data were expressed in terms of means and standard deviations. The visual assessment results by the Snellen chart were converted to a diopter unit (D) to make it comparable to other studies [8]. In the study, Myopia was defined as the spherical equivalent (SE) of ≤ − 0.5 D. We used a binomial statistical test to get a prevalence of confidence interval (CI) of 95%. In regard to risk factors, three groups (low, moderate, and high risk) were established for the following risk factors (family history, near activity, outdoor activity) [9]. Participants were categorized into myopic and non-myopic groups and a comparison was done between these groups in terms of sociodemographic and risk factors of myopia using the chi-square statistical test and results were considered statistically significant at p ≤ 0.05.

Ethical clearance was obtained from King Abdullah hospital in Bisha and agreements of parents on informed consent, all data collected were anonymous and confidential that were used for research purposes only.

## Results

A total of 330 male students were involved in the study (92.4% response rate) aged from 10 to 13 years with a mean age of 11.29 ± 0.97. Nearly 143 (43.3%) were in the sixth grade. The majority of students 284 (86.3%) were of Saudi nationality (Table 1).

**Table 1:** Sociodemographic data of students and their association with myopia. N= number of participants

Sociodemographic (N)	Myopia prevalence	P value
Age		P = 0.018
10 years (79)	27.16%	
11 years (116)	27.02%	
12 years (92)	40.42%	
School grade		P = 0.001
Fourth grade (85)	25.28%	
Fifth grade (102)	26.04%	
Sixth grade (143)	42.65%	
Nationality		P = 0.060
Saudi (284)	34.50%	
Non-Saudi (46)	22.22%	

The overall prevalence of myopia among students was 108 (32.7%, 95% CI: 27.7-38.1%). The mean spherical equivalent (SE) of participants was - 0.25 ± 0.60 D (Table 2). Myopia prevalence was noticed to increase progressively with age and school grade (p = 0.018). In addition, the prevalence tended to increase with school grades, from 25.2% in fourth grade to 42.6% in sixth grade (p = 0.001). Among all students around 27 (8%) were diagnosed with myopia and wearing eyeglasses on eye assessment. However, 77% of them had abnormal visual assessments and were wearing under-corrected eyeglasses with a mean SE of - 0.75.

**Table 2:** The prevalence of myopia and grades of spherical equivalent. SE = Spherical Equivalent, D = Diopter unit

SE	Percentage %
0.00 D (normal)	56.1%
− 0.25 D	21.65%
− 0.5 D	11%
− 0.75 D	5.6%
≤ − 1 D	5.25%

In regard to the duration of near activities, participants were allocated into three groups: low risk (less than two hours), moderate risk (between two to three hours), and high risk (more than three hours). Around 12% of students had high risk (more than 2.7 hours) and spent a lot of time carrying out near activities this was significantly associated with the presence of myopia (p ≤ 0.001) (Table 3). Children who spent more than 50% of their near activity time on electronic devices tend to have myopia more than other students (p = 0.001). Nearly a quarter of individuals 81 (24.5%) have either one or both parents with myopia. However, this was not associated significantly with the development of myopia among the students (p = 0.175). For the time spent on outdoor activity, it was divided into three groups also a low risk (more than three hours), moderate risk (between hours two to three hours), and high risk (less than two hours). Those who had long-time outdoors had the lowest prevalence of myopia (16%) this was statistically significant (p ≤ 0.001). Only 33 (10%) individuals do a regular screening for their eyes.

**Table 3:** Risk factors of developing myopia and the association with myopia prevalence.

Risk factors	Myopia prevalence	P value
Family history		P = 0.175
Low risk (neither suffers of myopia)	44.3%	
Moderate risk (one suffers from myopia)	42.8%	
High risk (both suffer of myopia)	33.3%	
Time spent doing near activities		P ≤ 0.001
Low risk (less than 1.6 hours)	31.4%	
Moderate risk (between 1.6-2.7 hours)	24.2%	
High risk (more than 2.7 hours)	58.1%	
Time spent outdoors		P ≤ 0.001
Low risk (more than 3 hours)	16.1%	
Moderate risk (between 2-3 hours)	47.8%	
High risk (less than 2 hours)	26.2%	

## Discussion

This cross-sectional study evaluated the prevalence of myopia and it is associated risk factors among primary school children aged 10-13 years old in Bisha, located in the southern region of Saudi Arabia. Which included 330 male students from five different schools in Bisha.

In our study, the prevalence of myopia was 32.7% which is comparable to previous studies in China (36.9%) [10], Nigeria (29.5%) [11], and Saudi Arabia (33.28%) [7]. But was higher than studies from Spain (18%) [12], Vietnam (14.2%) [13], and studies from Saudi Arabia (5.8%, 9.0 %, 4.5%) [14,15,16]. However, it was lower than previous reports in China (63%), (75.35%) [17,18], and India (39.6%) [6]. We found in this study that the prevalence of myopia increases with age which was similar to previous articles in Spain [12], Vietnam [13], and Saudi Arabia [7]. These differences might be attributed to variation in genetic suitability that varies according to race and cultural settings. 

In regard to risk factors of myopia, in our study students who spent long hours doing near activities are at high risk of developing myopia and this was similar to studies in Jordan [19], and Spain [12]. However, other articles showed that the long duration of near activity is not considered a risk factor to develop myopia in China [20], Singapore, and Sydney [21].

In this study, we found that children spending a long time doing outdoor activities are at low risk of having myopia. This result was similar to a previous study that aimed specifically to assess the relationship between outdoor activity and the prevalence of myopia [22]. On another hand, a study in Spain [12] showed that there is no significant association between the outdoors and the prevalence of myopia. In this study, the history of having either one or both parents with myopia is not significantly associated with the prevalence of myopia. On the contrary, studies from Spain [12], Jordan [19], and Vietnam [13].

There are some limitations to be mentioned, this study didn’t include female students due to the fact that the data collection process was on a health campaign provided only to boys' schools due to a lack of female staff. But, this can be overcome in the next project and campaign. Children were assessed by Snellen chart only not with the autorefractor, this was due to an insufficient of resources and sponsorship to the campaign. However, students with abnormal eye results were referred to an optometrist to evaluate the eye and prescribe suitable eyeglasses.

## Conclusions

Our study showed that the prevalence of myopia among primary school male students is considerably high In comparison to national studies in Saudi. Myopia prevalence tended to increase with age, school grades and spending a long time on near activities. Therefore, it is necessary to make a future screening program plan in order to minimize the prevalence and progression of myopia.
